# Factors influencing completion of multi-dose vaccine schedules in adolescents: a systematic review

**DOI:** 10.1186/s12889-016-2845-z

**Published:** 2016-02-19

**Authors:** K. E. Gallagher, E. Kadokura, L. O. Eckert, S. Miyake, S. Mounier-Jack, M. Aldea, D. A. Ross, D. Watson-Jones

**Affiliations:** Clinical Research Department, Faculty of Infectious Tropical Diseases, London School of Hygiene and Tropical Medicine, Keppel Street, London, WC1E 7HT UK; Mwanza Intervention Trials Unit, The National Institute for Medical Research Mwanza Campus, PO Box 11936, Mwanza, Tanzania; Department of Epidemiology, University of Washington, 1959 NE Pacific Street, Health Sciences Building F-250, Box 357236, Seattle, WA 98195-7236 USA; Departments of Obstetrics and Gynaecology and Global Health, University of Washington, 1959 NE Pacific Street, Health Sciences Building F-250, Box 357236, Seattle, WA 98195-7236 USA; Department of Global Health and Development, Faculty of Public Health and Policy, London School of Hygiene and Tropical Medicine, 15-17 Tavistock Place, London, WC1H 9SH UK; Infections and Cancer Unit, Cancer Epidemiology Research Programme, Catalan Institute of Oncology, Av. Gran Via de l’Hospitalet 199-203, Hospitalet de Llobregat, 08908 Barcelona, Spain; Bellvitge Institute of Biomedical Research (IDIBELL), Barcelona, Spain; MRC Tropical Epidemiology Group, Faculty of Epidemiology and Population Health, London school of Hygiene and Tropical Medicine, Keppel Street, London, WC1E 7HT UK

**Keywords:** Vaccines and immunization, Immunization programmes, Vaccination completion, Barriers, Adolescent health services

## Abstract

**Background:**

Completion of multiple dose vaccine schedules is crucial to ensure a protective immune response, and maximise vaccine cost-effectiveness. While barriers and facilitators to vaccine uptake have recently been reviewed, there is no comprehensive review of factors influencing subsequent adherence or completion, which is key to achieving vaccine effectiveness. This study identifies and summarises the literature on factors affecting completion of multi-dose vaccine schedules by adolescents.

**Methods:**

Ten online databases and four websites were searched (February 2014). Studies with analysis of factors predicting completion of multi-dose vaccines were included. Study participants within 9–19 years of age were included in the review. The defined outcome was completion of the vaccine series within 1 year among those who received the first dose.

**Results:**

Overall, 6159 abstracts were screened, and 502 full texts were reviewed. Sixty one studies were eligible for this review. All except two were set in high-income countries. Included studies evaluated human papillomavirus vaccine, hepatitis A, hepatitis B, and varicella vaccines. Reported vaccine completion rates, among those who initiated vaccination, ranged from 27 % to over 90 %. Minority racial or ethnic groups and inadequate health insurance coverage were risk factors for low completion, irrespective of initiation rates. Parental healthcare seeking behaviour was positively associated with completion. Vaccine delivery in schools was associated with higher completion than delivery in the community or health facilities. Gender, prior healthcare use and socio-economic status rarely remained significant risks or protective factors in multivariate analysis.

**Conclusions:**

Almost all studies investigating factors affecting completion have been carried out in developed countries and investigate a limited range of variables. Increased understanding of barriers to completion in adolescents will be invaluable to future new vaccine introductions and the further development of an adolescent health platform.

PROSPERO reg# CRD42014006765.

**Electronic supplementary material:**

The online version of this article (doi:10.1186/s12889-016-2845-z) contains supplementary material, which is available to authorized users.

## Background

In the past decade there has been an increase in the number of new vaccines licensed worldwide [1, 2] and in the accessible funding for vaccine introduction to low-resource settings through the founding of Gavi, The Vaccine Alliance, in 2000 [3]. Multi-dose vaccines in the WHO recommended immunization schedule for adolescents are listed in Table 1; WHO defines adolescence as age 10–19 years inclusive. National vaccine schedules can depart from WHO recommendations, e.g. 2 doses of hepatitis A and meningococcal conjugate vaccines (MCV4) are offered to adolescents in the USA [4]. Although recommended for administration at birth, hepatitis B vaccine (HBV) is routinely offered to older children and adolescents if not previously immunised [5]. In settings where varicella is seen as a public health priority WHO recommends 2 doses of varicella vaccine, with the first dose at 12–18 months and up to 4 month interval between doses [6, 7]. The most recently licensed multi-dose vaccines are the human papillomavirus (HPV) vaccines. In 2014, HPV vaccine recommendations were revised by WHO SAGE from a schedule of 3 doses [8], to 2 doses at a 6 month interval in girls less than or equal to 15 years of age [9, 10] based on evidence of non-inferior immunogenicity [11, 12].

**Table 1:** WHO recommended vaccine schedule for adolescents^a, b^. Caption: the WHO recommended schedule of vaccines for adolescents (10–19 years of age inclusive), if not given prior to age 10 years

Recommended vaccines for all adolescents	Adolescent dosage	Licensed age
Tetanus, diphtheria, pertussis	3 doses Tdap^a^ & Td Booster	Infant onwards
Human papillomavirus	2 doses if ≤15 years	≥9 years
	3 doses thereafter	
Meningococcal conjugate	MenA: 1 dose	Infant onwards
	MenC: 1 dose	
	MCV4: 1 dose	
Influenza	1 dose Yearly booster	≥9 years
Hepatitis A	1 dose	Infant onwards
Hepatitis B	3 doses^a^	Infant onwards
Measles, Mumps, Rubella	1 dose^a^	Infant onwards
Recommended in at-risk areas	Adolescent dosage	Licensed age
Tick borne encephalitis	3 doses	Infant onwards
Japanese encephalitis	1 dose	Infant onwards
Typhoid	Vi polysaccharide: 1 dose	Infant onwards
	Ty21a live oral vaccine: 3–4 doses	
	Booster 3–7 years after primary series	
Cholera	Dukoral, Shanchol & mORCVAX: 2 doses booster every 2^nd^ yr	≥2 years
Rabies	3 doses	Infant onwards
Varicella	2 doses	≥9–12 months

^a^Recommended schedule if not administered prior to age 10 years

^b^World Health Organization. WHO recommendations for routine immunization - summary tables - http://www.who.int/immunization/policy/immunization_tables/en/ 2014

At present, evidence suggests multiple doses of HBV, HPV, and varicella vaccines are needed for efficacious protection against disease in adolescents [5, 9, 13, 14]. However; completion of the vaccine dose series, defined as receipt of the final dose within 1 year of the first dose, has proven challenging in some settings. Completion rates of HPV vaccine were lower than 30 % in the first years of introduction in the USA [15, 16]. Addressing specific difficulties in administering vaccines to adolescents will be invaluable for implementation of future adolescent vaccines and further developing adolescent health services.

The currently available reviews of factors influencing completion focus solely on selected developed countries [1, 17–19], have non-systematic searches [20, 21] or need updating [22]. This systematic review describes factors which have been investigated for their effect on multi-dose vaccine adherence in adolescents to aid development of interventions to improve adherence.

## Methods

### Search strategy

A comprehensive set of search terms was built around: 1) childhood/adolescence; 2) vaccination/immunisation; 3) adherence/completion. Articles with at least one term from each topic were identified. Search terms were informed by the Cochrane Child Health Group recommended terms for adolescents or school children [23] and included international spelling variations (Additional file 1: Table S1). Multi-dose vaccines administered to adolescents were identified through the Centers for Disease, Control, and Prevention (CDC) [6, 24], and the WHO list of prequalified vaccines [25]; however, search terms were not limited to these vaccines.

Medline, Embase, Global Health (Ovid SP), Popline, Web of Science, Africa Portal, Africa-wide information, ADOLEC, Cochrane, Open Grey databases, and PATH, Gavi, and WHO websites were searched in February 2014. No publication date restriction was set. Publications, abstracts and conference proceeding were eligible for inclusion. All texts were collated and reviewed using Endnote X7 (Thompson Reuters); automated and manual de-duplication was performed.

### Inclusion criteria

**Fig. 1:**
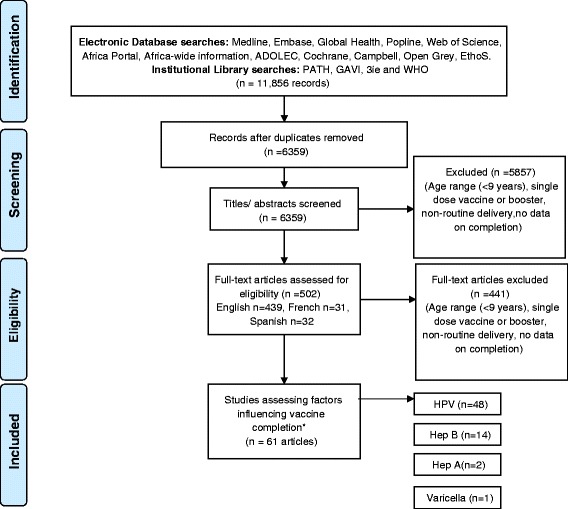
Systematic review flow diagram. Caption: the PRISMA flow diagram for the systematic review detailing the database searches, the number of abstracts screened and the full texts retrieved. ^*^Some articles analysed >1 vaccine

Inclusion criteria for consideration of studies were outlined in a protocol a-priori as per PRISMA guidelines [26] (Table 2). The title and/or abstract of each article were reviewed in the first instance by a single reviewer (KG). Modelling studies, immunogenicity/efficacy trials were excluded. Two reviewers (KG, SM/EK) screened the abstracts. Any study including a vaccine for which more than one dose was administered to persons 9–19 years old in a routine setting within 1 year was considered for inclusion. The WHO definition of adolescent (10–19 years old) was widened to include 9 year olds to include all participants in HPV vaccine studies (WHO recommended for 9–13 year olds). Inclusion criteria were independently applied to full texts by 2 authors (KG, SM/EK) (Fig. 1) [26].

**Table 2:** Study inclusion criteria. Caption: abstracts and full texts were screened independently by two authors using the following criteria

Study definitions and characteristics	Inclusion criteria: studies investigating factors governing adherence
Study population	Any child/adolescent 9–19 years old, recruited from the community or a cohort of vaccinees, care-givers or care-providers
Geographical setting	No restriction
Vaccine	Any vaccine administered to the study population in a schedule including more than one dose within the same year
Vaccine delivery	Routine vaccine delivery; studies excluded if an outbreak/campaign setting/non-routine delivery
Outcome	Completion or non-completion of (or ‘adherence to’) the intended multi-dose vaccine schedule within 1 year of follow-up
Comparison	Individuals or groups who initiated vaccination (i.e. received dose 1), and completed the vaccine series (i.e. received the final dose) within 1 year, compared to those who initiated the vaccine series but did not receive the final dose within 1 year.
Exposure	Any characteristics of individuals, communities, or programmatic or contextual factors investigated for an association with adherence/completion
Study design	Any study design with data on and analysis of factors predicting completion of a multi-dose vaccine in routine settings
Data	Some estimate of the completion rate achieved must be available

### Data extraction

Data were extracted by 2 authors (KG, EK) into separate forms on Microsoft Excel 2010. Article selection and data extraction discrepancies were resolved through discussion. Data on the study population, setting, vaccine, rates of completion and the factors investigated were extracted alongside descriptive, univariate and multivariate results, as applicable.

### Assessment of bias

An assessment of bias was recorded on the data extraction form for each study using criteria outlined in the Cochrane tool for assessment of bias in intervention and epidemiological studies [27]. Selection bias and information bias were assessed alongside the potential for confounding.

### Data synthesis

Heterogeneity in study methods, population, context and classification of exposure variables, led to a descriptive synthesis. Groups which initiated and completed the recommended vaccine schedule within 1 year were compared to non-completers who only initiated.

## Results

Of the 502 full texts reviewed (Fig. 1), 61 articles were eligible for inclusion (Table 3). Included articles reported completion rates for HPV (3-dose completion ranged from 27 % [15] to over 90 % [28]), HBV (27 % completion before a school mandate was introduced in California [29] to 95 % in a school-based programme in Canada [30]), varicella and hepatitis A vaccine (HAV). In the USA, the two dose series of varicella vaccine was completed within 1 year in 35.9 % of adolescents and 2 doses of HAV were completed in 40–48 % [31]. Searches returned no articles on completion of conjugate meningococcal vaccine, despite a multi-dose policy in the USA [32]. For the purposes of this review we have focused on results from multivariate analyses or qualitative findings. Data availability by country and vaccine is displayed in Table 4.

**Table 3:** Summary of included studies. Caption: a summary of the studies included in the review, including details of sample size, the source of the sample, year of data collection, vaccine investigated, target age group, delivery strategy, completion rate attained and factors investigated to influence completion

Author; date	Sample size	Country; source of sample	Year of data collection	Vaccine	Vaccine target age group	Vaccine delivery strategy	Completion rate	Factors investigated to influence completion
Bednarczyk, R; et al. 2011	588	USA. New York state University health clinics and classrooms: self-report questionnaire.	2010	HPV	11–12 (with catch up to 26)	Available at routine healthcare provider	79 %	Qualitative interviews
Carlos, R; et al. 2010	232	USA. Mailed questionnaire to attendees of breast and cervical cancer screening clinics (maternal report).	2010	HPV	11–12 (with catch up to 26)	Available at routine healthcare provider	19 %	Race
Cassidy, W; Mahoney, F. 1995	654	USA. School and administrative data.	1994-5	HBV	School grades 6–8	School-based clinic	82 %	Gender, race
Chao, C; Slezak, J; et al. 2009	18,275	USA. Electronic health records from KPSC managed care organisation.	2006-8	HPV	9–26	Available at routine healthcare provider	43 %	Maternal characteristics: history of at least 1 Pap test in the past 3 years, history of abnormal pap test result, history of genital warts/ other STIs, SES (neighbourhood median household income, neighbourhood average adult education)
Chao, C; Velicer, C; et al. 2009	34,193	USA. Electronic health records from KPSC managed care organisation.	2006-8	HPV	9–26	Available at routine healthcare provider	41 %	age, race, socioeconomic status (census block neighbourhood statistics, medicaid eligibility), provider characteristics, health care utilization, women's health related conditions, chronic illness
Chou, B; et al. 2011	1413	USA. Electronic health records from ambulatory care clinics (4) associated with a University.	2007-8	HPV	11–12 (with catch up to 26)	Available at routine healthcare provider	33 %	Age, insurance (private/public), provider characteristics (location, practice type (pediatrics, gynaecology or family practice)), race (White, African American, Hispanic).
Cleves, M. 1998	520	USA. Medical records.	1995-6	HBV	11–19	Available at routine healthcare provider	33 %	Age, race, insurance, sexual activity, risk behaviour (drug use)
Cook, R; et al. 2010	11,986	USA. Medicaid administrative data.	2006-8	HPV	9–20	Available at routine healthcare provider	27 %	Age, race, provider of first shot, insurance (months of medicaid enrollment), sexual activity.
Crosby, R; et al. 2011	209	USA. University of Kentucky, rural community college and rural health clinic attendees	2007-8	HPV	9–26	Available at routine healthcare provider	56 % urban	Geography (rural/urban location)
							10 % rural	
Deeks, S; Johnson, I. 1998	39,935	Canada. Administrative data from Health units (7), Greater Toronto Area	1994-5	HBV	School Grade 7	School-based delivery	95 %	School characteristics, knowledge/ education/mobilisation
Dempsey, A; et al. 2010	2625	USA. Health records from 20 university-affiliated health clinics, Michigan	2007-8	HPV	9–26	Available at routine healthcare provider	15 %	Age, insurance, race
Dempsey, A; et al. 2012	1714	USA. Health records from 20 university-affiliated health clinics, Michigan	2008-9	HPV	9–26	Available at routine healthcare provider	53 %	Age, insurance, race
Dorell, C; et al. 2011	18,228	USA. Stratified, national, probability sample of households (NIS-teen survey)	2008-10	HPV	9–26	Available at routine healthcare provider	53 %	Age, insurance, health care utilization, household income, maternal education level, maternal age, maternal marital status, race, geography/ location
Fournier, M; et al. 2013	1404	USA. Electronic medical records from 2 primary care clinics	2007-12	HPV	11–12 (catch up to 26)	Available at routine healthcare provider	40 %	Insurance, race, health care utilization (other vaccines)
Ganry, O; et al. 2013		France. Electronic records of the Regime General Insurance (for workers), the RSI (for self-employed) and the RSA (agricultural occupations)	2009-10	HPV	14 (with a catch up to 23)	Available at routine healthcare provider	39 %	Age, insurance, provider characteristics.
					(recently revised to 11–14)			
Gold, R; et al. 2013	786	USA. Electronic medical records from an integrated managed care organisation	2008	HPV	11–12 (catch up to 26)	Available at routine healthcare provider		Socioeconomic status; health care utilization; provider characteristics; vaccine delivery concomitant with first dose; experiences at the first visit, challenges to making or keeping the index appointment; Knowledge and attitudes about HPV; adverse events.
Gold, R; et al. 2011	450	USA. Administrative data from 19 school-based health centres	2007-8	HPV	11–12 (catch up to 26)	Available at routine healthcare provider	51 %	Age, race and insurance status
Gonzalez, I; et al. 2002	79,357	USA. Electronic Data from 3 Health Management Organisations	1998	HBV	11–12	Available at routine healthcare provider	73 %, 67 %, 45 %	Provider characteristics (Health care organisation)
Harper, D; et al. 2013	2422	USA. Electronic records from a safety net health care system Kansas	2006-09	HPV	11–12 (catch up to 26)	Available at routine healthcare provider	42 %	Age, race, concomitant (visit type for first dose)
Hirth, J; et al. 2012	271,976	USA. Electronic records of a private insurance company	2006-10	HPV	11–12 (catch up to 26)	Available at routine healthcare provider	38 %	Age, provider type, time
Kester, L; et al. 2011	500	USA. Knowledge networks coordinated survey (nationally representative)	2010	HPV	11–12 (catch up to 26)	Available at routine healthcare provider	81 %	Race, insurance, maternal education, maternal relationship status, maternal history of HPV related condition, geography.
Kouyoumdjian, F; Bailowitz, A. 2011	18	USA. Baltimore city health department self report interviews	2007-9	HPV	11–12 (catch up to 26)	Available at routine healthcare provider	9.80 %	Geography (access), adverse events, qualitative reasons (convenience, knowledge, pain/discomfort)
Ladner, J; et al. 2012	87580	Multi-country (7 low resource countries). Administrative data from Gardasil Access Programme grantee countries	2009-11	HPV	Bhutan, Bolivia, Haiti, Nepal: 9–13	School based, health centre based or mixed strategies	Bhutan: 88 %, Bolivia: 96 %; Cambodia: 95 %; Cameroon 83 %, Haiti 76 %, Lesotho 93 %; Nepal 99 %.	Delivery strategy (school-based, health facility model, mixed model)
					Cambodia: 11–18			
					Cameroon: 9–18			
					Lesotho: 10–18			
Lancman, H; et al. 2000	3 centres	USA. Administrative data from 2 school based health centres and one adolescent health clinic	1997-98	HBV	11 and above	Available at school based health centres and routine providers	24 %, 29 %, 76 %	Health centre characteristics
Laz, T; et al. 2012	11,277	USA. Household questionnaire sent to parents	2010	HPV	11–12 (catch up to 26)	Available at routine healthcare provider	49 %	Age, parental education, insurance, race, parental income.
Lions, C; et al. 2013	105,327	France. National Insurance Reimbursement database	2007-8	HPV	11–14 (catch up to 19)	Available at routine healthcare provider	64.10 %	Age, insurance, geography, medical utilization
Macdonald, V; et al. 2007	2471	Australia. Health centre records from a primary health care centre, Sydney (high risk population)	1992-2003	HBV	High risk adolescent of any age	Available at routine healthcare provider and specialist clinics	21 %	Age, gender, race (aboriginal), risk behaviour (IDU, sex worker, hep.C status) length of contact with the health centre, accelerated versus normal schedule.
Moore, G; et al. 2010	209	USA. Medical records of community health clinic attendees	Unknown	HPV	11–12 (catch up to 26)	Available at routine healthcare provider	28 %	Attitudes and believes including perceptions of risk, peer experience of HPV vaccine, experience of cancer
Nelson, J C; et al; 2009	590445	USA. Vaccine Safety Datalink population (MCO registry)	1996-2004	Varicella, HAV, HBV	9–17	Available at routine healthcare provider	Varicella: 35.9 %; HAV: 48.4 % (age 9–12), 40.3 % (age 13–17); HBV: 63.4 % (age 9–12), 45.1 % (age 13–17).	Age, provider site, gender, length of MCO enrollment, year of first dose, utilization of medical visits in year prior to dose 1
Sinka, K; et al. 2014	86769	UK. The Child Health System database and the Scottish Immunisation Recall System	2008-11	HPV	12–13 (catch up for 13–17)	School-based (catch up included supply of vaccine at primary health care)	Year 1: 89.4 %	Scottish Index of Multiple Deprivation (SIMD)
							Year 2: 86.9 %	
							Year 3: 81 %	
Markovitz, A; et al. 2014	13,709	USA. Immunization registry, Michigan residents continuously enrolled with a PPO	2006-11	HPV	11–12 (catch up to 26)	Available at routine healthcare provider	22 %	Maternal preventive care utilization (Pap testing, mammograms, primary care office visits), age, race, household education, household income, maternal age.
Middleman, A. 2004	11,500	USA. School data	1998-2000	HBV	School grades 5–6	School-based	72 %	Insurance, race and gender
Middleman, A B; et al. 1996	826	USA. Medical records from an adolescent health clinic	Unknown	HBV	Any adolescent attending the clinic	Available at routine healthcare provider	23 %	Socio-demographics, risk behaviors (for hepatitis B), medication use, chronic illnesses, and experience, knowledge and attitudes about hepatitis B and the immunization
Middleman, A B; et al. 1999	943	USA. Questionnaires distributed at hospital and school based clinics	1994-5	HBV	Any adolescent attending the clinics	Available at routine healthcare provider and school based clinic	47.6 % (Clinic); 41.7 % (School-based clinic)	Race, insurance, residential zip code, risk factors for acquiring hepatitis B, risk behaviors (cigarette and substance use), and academic achievement, chronic illness, healthcare utilization, knowledge about hepatitis B and the vaccination, family history of hepatitis B vaccination, travel time, and mode of transportation to the clinic.
Monnat, S; Wallington, S. 2013	4,776	USA. Behavioral Risk Factor Surveillance System Survey data in 10 territories	2008-10	HPV	11–12 (catch up to 26)	Available at routine healthcare provider	14 %	Mother’s history of cervical screening (Pap test).
MooreCaldwell, S; et al. 1997	174	USA. Medical records from a university adolescent clinic and junior-senior private high school clinic	1992-3	HBV	Any adolescent attending the clinics	Available at routine healthcare provider at school based clinics	89 %	Adolescent and parent knowledge of hepatitis B, perceived risk.
Moss, J L; et al. 2013	105,121	USA. North Carolina Immunization Registry	Not available	HPV	11–12 (catch up to 26)	Available at routine healthcare provider	28 %	Gender ratio, race, provider specialty and adolescent patient load, reminder/recall system, time to documentation in NCIR, computers per clinic, age of vaccine recommendation (Tdap, Meningococcal, HPV)
Musto, R; et al. 2013	35,592	Canada. Calgary zone Public Health vaccination database	2008-11	HPV	Grades 5 and 9	School-based programme and available at community public health clinics	75 % (School-based);	In-school vs community health clinic delivery model, socioeconomic status, school provider type, history of HBV.
							36 % (community)	
Neubrand, T; et al. 2009	352	USA. Medical records review from two different sites	2007-8	HPV	11–12 (catch up to 26)	Available at routine healthcare provider	58 %	Age, race, insurance (private vs Medicaid/Child Health Insurance Program [CHIP]), and distance from home to the clinic, sexual activity prior to initiation of the series, history of an STI, cervical screening history within 3 years of vaccine initiation, reason for clinic visit
Niccolai, L; et al. 2011	7606	USA. NIS-Teen survey: Random digit dialing household survey.	2008-9	HPV	11–12 (catch up to 26)	Available at routine healthcare provider	55 %	Race, socioeconomic status, age, maternal characteristics, insurance, healthcare utilization, geography (region) and year.
Perkins, R B; et al. 2012	7702	USA. Electronic medical records from Boston Medical/ community health centers	2007-8	HPV	11–12 (catch up to 26)	Available at routine healthcare provider	20 %	Age, location, number of clinic visits in study period, race, risk behaviour (documentation of STI or alcohol use), history of meningococcal or tdap booster vaccine.
Pruitt, C N; et al. 2013	978	USA. Rochester Epidemiology Project records (REP) from medical records	2006-9	HPV	11–12 (catch up to 26)	Available at routine healthcare provider	Somali girls: 52 %; white/non-Hispanic: 72 %	Somali ethnicity
Rahman, et al. 2013	2632	USA. Data from Behavioral Risk Factor Surveillance System Telephone survey	2008-10	HPV	11–12 (catch up to 26)	Available at routine healthcare provider	17 %	Geography/ location
Reiter, P L; et al. 2009	229	USA. Telephone survey, North Carolina.	2007-8	HPV	11–12 (catch up to 26)	Available at routine healthcare provider	83 %	Adverse events/ reported pain from HPV vaccination
Reiter, P; et al. 2013	1951	USA. NIS-Teen survey: Random digit telephone survey	2008-10	HPV	11–12 (catch up to 26)	Available at routine healthcare provider	28 %	Age, race, healthcare utilization in last year, insurance, maternal characteristics, knowledge of HPV, provider recommendation, socioeconomic status.
Rouzier, R; Giordanella, J. 2010	77,744	France. CPAM social security database	2007-8	HPV	14 (Catch up to 23)	Available at routine healthcare provider	43 %	Age, provider (general practitioner vs. gynecologist)
Rubin, R; et al. 2012	10,821	USA, Administrative reimbursement data and medical records from medical group practices	2006-10	HPV	11–12 (catch up to 26)	Available at routine healthcare provider	27 %	Pre-existing STD, age, provider medical department
Sakou, I I; et al. 2011	1005	Greece. Convenience sample of Adolescent Health Unit attendees	2009	HBV, HAV, HPV	HPV: 12–15; HAV, HBV: catch up 11–18	Available at routine healthcare provider	Not reported	Gender, race/ nationality, parental education, family status
Schluterman, N H; et al. 2011	8069	USA. Database of the University of Maryland Medical Center (UMMC)	2006-10	HPV	11–12 (catch up to 26)	Available at routine healthcare provider	11 %	Race, insurance status (publicly funded, private, or none), age (9–13, 14–17, or 18–26 years), and place of residence (urban or suburban Baltimore).
Schmidt, M A; et al. 2013	311213	USA. Administrative data from vaccination sites	2006-11	HPV	11–12 (catch up to 26)	Available at routine healthcare provider	42 %	Age, calendar year
Schmitt, K; Thompson, D. 2013	n/a	USA. Statewide Immunization Registry	2001-11	HPV	11–12 (catch up to 26)	Available at routine healthcare provider	52 %	Age, insurance, provider type, race
Seid, M; et al. 2001	800	USA. Survey to parents of children at 5 Schools, San Diego	1998	HBV	11–12	Available at routine healthcare provider	27 %	Provider, school based clinics, school socioeconomic status, home language, race, insurance, health care utilization, heard about mandatory vaccination from health care provider.
Smith, L M; et al. 2011	2519	Canada. Universal health insurance program database.	2007-10	HPV	School grade 8	School-based	86 %	Age, parental income, and place of residence, vaccination history, health services utilisation, medical history.
Tan, W; et al. 2011	138823	USA. NCIR immunisation registry	2006-2009	HPV	11–12 (catch up to 26)	Available at routine healthcare provider	55 %	Race, age, county of residence, provider clinic type, insurance.
Teplow-Phipps, R; et al. 2014	1,494767	USA. Citywide Immunization Registry (CIR), New York City	2005-12	HPV	11–12 (catch up to 26)	Available at routine healthcare provider	38.4 % (females)	Age, gender, insurance, clinic specific variables: provider practice-type, number of Tdap vaccines reported (proxy for practice size), and socioeconomic status of practice location.
							35.7 % (males)	
Tracy, J K; et al. 2010	9658	USA. Clinical data repository at the University of Maryland Medical Center	2006-10	HPV	11–12 (catch up to 26)	Available at routine healthcare provider	31 %	Age, race.
Tung, C S; Middleman, A B. 2005	8918	USA, Data from 75 schools participating in HBII (Hep B immunization initiative).	1999-2000	HBV	13–15	School-based	59 %	Publicity/promotion, packet distribution, return of forms, ratio of students to clinic, provider characteristics
Verdenius, I; et al. 2013	1563	USA. Electronic medical records	2006-9	HPV	11–12 (catch up to 26)	Available at routine healthcare provider	32 %	Age, type of health visit, healthcare utilization, concomitant healthcare delivery.
Widdice, L E; et al. 2011	3297	USA. Review of medical records from academic medical center	2006-8	HPV	11–12 (catch up to 26)	Available at routine healthcare provider	28 %	Age, race, insurance, healthcare utilization (DMPA), clinic location, time period of vaccine series initiation
LaMontagne, D; et al. 2011	7269	Peru, India, Uganda, Vietnam. Population based household survey	2008-10	HPV	Peru: grade 5; Uganda: grade 5 or age 10; Vietnam: grade 6 or age 11; India: 10–14.	School-based or health centre based in all 4 countries	Not reported	Delivery Strategy

**Table 4:** Data available on factors investigated across countries and vaccines

Factor investigated	Countries (Number of studies with multivariate analyses)	Vaccine			
		HPV	HBV	HAV	Varicella
Age	USA (17), Canada (1), France (1), Australia (1)	✓			
Race	USA (16), Australia (1), Greece (1)	✓	✓	✓	
Insurance	USA (15), France (1)	✓	✓	✓	✓
Gender	Australia (1), USA (2)		✓		✓
Socio-economic status	USA (11), Canada (1), UK (1), France (1)	✓	✓		
Healthcare utilization	USA (14), France (1), Australia (1), Canada (1)	✓	✓		✓
Vaccine knowledge	USA (3)	✓	✓		
Adverse events	USA (3)	✓			
Risk behaviour	USA (3), Australia (1)	✓	✓		
Concomitant healthcare	USA (3)	✓			
Access	USA (2)	✓	✓		
Maternal characteristics	Pap smear history – USA (3)	✓		✓	
	Education – USA (7), Greece (1)				

### Individual level factors

#### Age

The association between age and completion was investigated in 31 articles. Multivariate analyses of at least 2 age categories within the age range of 9–19 years were conducted in 20 studies, in the USA (*n* = 17), Canada [33], France [34], and Australia [35]. Age recommendations vary across countries; results must be interpreted in context.

There is some evidence that completion rates decrease as age of vaccine initiation increases for HPV vaccine, HAV, and HBV [15, 31, 36–38]. In the USA, the HPV vaccine recommended age range is between 11 and 26 years; five studies state similar results among Medicaid enrolees, adjusting for insurance, race, region and year, 17 year olds were 0.84 times less likely to complete HPV vaccine compared to 11 year olds (95 % CI 0.74–0.95) [15]. Among attendees of an urban hospital, in adjusted analyses, 14–17 year olds had 0.71 the odds of completion HPV vaccine when compared to 9–13 years olds (95 % CI 0.59–0.98) [37].

In the USA five further studies found no association [33, 39–43] and two studies report the converse association, increased likelihood of completion with age between 13 and 17 years controlling for year, race, insurance status; this perhaps reflects the perception that it was an ‘STI vaccine’ in 2007–8 [44, 45]. No association between age and HPV vaccine completion was found in multivariate analyses in Canada although only one school grade was targeted [33, 39–43].

### Race

Racial or ethnic identity was analysed in 31 studies from the USA, Australia and Greece; 18 conducted multivariate analyses. Analysis of >100,000 women in North Carolina adjusted for location, clinic, insurance, and age found Black (aOR 0.55; 95 % CI 0.53–0.56), American Indian or Alaskan (aOR 0.68; 0.61–0.77) and Hispanic (aOR 0.75; 0.72–0.79) women had 25–45 % lower likelihood of completion compared to White women [43]. Race was the only significant predictor of completion in the NIS-Teen household survey in USA [44]. Ten additional large database studies in the USA with multivariate analyses corroborate this association for both HPV and HBV vaccines [15, 16, 36, 37, 39, 40, 42, 43, 46–48]. However, no association between race and completion was found in 5 studies when controlling for gender, insurance and health clinic characteristics [29, 38, 45, 49, 50]. Hispanic adolescents were underrepresented in one survey with a low response rate [29].

Greek non-nationals had lower completion rates (33 %) than nationals (60 %) for 2 doses of HAV [51]. In the northern territories of Australia, 3 dose coverage of HPV vaccine was lower in indigenous compared to non-indigenous groups (54 % vs. 64 %), but completion rates were the same (84 %) [52].

### Insurance

Many countries have supplied HPV vaccine free-of-charge. In the USA, although the vaccine was not initially eligible for reimbursement in some health insurance plans, after it was recommended by the Advisory Committee on Immunization Practices it was included in the Vaccines for Children (VFC) programme which provides for underinsured and uninsured children [1]. Insurance status was investigated as a risk factor in 25 articles, 16 conducted multivariate analyses (15 USA, 1 France). In 2011, insurance status remained a significant predictor of HPV series completion in the USA; those publicly insured (Medicaid) were 2.08 times (95 % CI 1.16–3.7) more likely to complete compared to those with no insurance; those privately insured were not significantly more likely to complete than those on public insurance (aOR 1.16; 95 % CI 0.97–1.38) controlling for age, race, contraception use [42]. The association between insurance status and completion was stronger in 2006–8 reflecting policy changes [16, 43]. In France completion rates were lower among recipients of complimentary social welfare compared to those with private insurance (aRR 0.88; 95 % CI 0.83–0.93) [34].

Longer enrolment on an insurance plan (>12 years) was associated with a 1–14 % increase in likelihood of completion of 2 doses of varicella vaccine; 9–12 % increased likelihood for HAV and 21–23 % for HBV in the vaccine safety database of almost 600,000 people in the USA between 1998 and 2004 [31] controlling for age, gender, healthcare utilisation and provider characteristics.

Across the USA there are substantial differences across states in beliefs, policy, and the rapidity of implementation of changes made at the national level. In Oregon state in 2008, HPV vaccine was offered free of charge and no difference was found in completion rates between publicly and privately insured participants [53]. In Maryland in 2006–10 private insurance was found to be a risk factor for non-completion compared to those publicly insured (aOR 0.76; 95 % CI 0.59–0.98), controlling for race and age [37]. No association in multivariate analyses was seen in 5 studies in the USA [29, 39, 40, 44, 50].

### Gender

Gender was assessed in seven articles; no correlation between completion of HBV and gender was seen in unadjusted results from Greece [51], nor in adjusted results in Australia [35]. In the USA, controlling for delivery site, age, insurance, year, chronic conditions and prior healthcare utilization, male gender was marginally associated with lower completion for varicella (aOR 0.93; 95 % CI 0.90–0.96), HAV (aOR 0.98; 0.97–0.99) and HBV (aOR 0.97; 0.96–0.98) [31]. Included studies did not report completion of HPV vaccine in boys, recommendations to vaccinate boys were issued in 2015 in the USA; however, clinics in the USA with higher female:male ratios obtained higher completion rates of HPV vaccine among females (aRR 2.16; 1.13–4.13) [49].

### Socio-economic status

Socio-economic status (SES) was analysed in studies in the USA (*n* = 14), Canada (*n* = 2), UK (*n* = 1) and France (*n* = 1); 14 conducted multivariate analysis. Median neighbourhood income and average adult education [54], parental income levels [39, 50, 55], household income [56] and poverty status [45] were not associated with completion in multivariate analyses.

Every 10,000USD rise in median neighbourhood income was associated with a 15 % increase in HBV completion (aRR 1.15; 95 % CI 1.06–1.25) [47] and a 1 % increased likelihood of HPV completion in 20,000 9–17 year old American girls (aRR 1.01; 1.01–1.02) [57]. Average census block education level was positively associated at a similar magnitude of effect (aRR 1.03; 1.02–1.05) [57]. Adolescent girls living below the federal poverty level were signifıcantly less likely to complete vaccination compared to adolescents with household incomes > $75,000 (aOR 0.76; 0.63–0.92) [44].

The effect of SES may differ by delivery strategy; in Canadian public schools with in-school HPV vaccine delivery, completion increased as SES decreased, in Catholic schools in which the pupils relied on community delivery, completion decreased as SES decreased [58]. A linear trend with the Scottish multiple index of deprivation was found with completion but not with initiation; however, the difference between the most and least deprived groups was small (8 %) and disappeared with the administration of a catch-up dose 1 year later [59]. Girls in Canada in 2007–8 living in lower income neighbourhoods were significantly less likely to complete HPV vaccine than girls living in middle income neighbourhoods (aOR 0.45; 0.28–0.72) [33]. In France compliance with the HPV vaccine schedule was lower in social welfare recipients compared to non-recipients (aRR 0.88, 0.83–0.93) [34].

### Healthcare utilization

History of health care utilization was inconsistently associated with completion. Seventeen articles from the USA, France and Australia analysed an individual’s prior use of health care (defined by receipt of other recommended vaccines, or the number of prior visits to a primary health care provider) and completion of a multi-dose series of varicella, HPV or HBV vaccines. In adjusted analyses in the USA, >10 visits to a health care provider in the last year was associated with 15 % increased likelihood of HPV vaccine completion and a 4–6 % increase in HBV completion [31]. Similar findings were reported in France where compliance with the HPV vaccine regimen was 10 % higher if a girl had >6 consultations with a family physician in the past year [34]. The magnitude of the effect is supported by reports of a 2 % increased likelihood of completing the HPV vaccine series with every primary care provider visit in the past year [36].

A further eight studies found no association between vaccine completion and the number of visits to a primary healthcare in the preceding 2 years [60], non-acute care in the year preceding initiation [29, 33, 39, 44, 55], previous prescriptions [47] or receipt of tetanus, diphtheria, and pertussis booster (Tdap) and meningococcal vaccines [38].

Recorded contraceptive use (DMPA) at any time in the medical records by HPV vaccine recipients was associated with a two-fold increase (95 % CI 1.72–2.47) in the odds of HPV vaccine completion [42]. In Canada, HBV vaccination conferred 16.9 times higher odds (95 % CI 14.8–19.2) for HPV vaccine completion in comparison with those who had not received HBV. However, the association could be confounded by the differing vaccination policies and delivery strategies by school [58]. In Australia in an area with a high risk population, including young sex workers and drug users, a shorter time interval (<2 weeks) between first contact with the health care provider and initiation of vaccine series correlated with better HBV completion [35].

### Vaccine related knowledge

Three American studies examined knowledge in relation to completion in multivariate analysis [45, 47, 55]. The ability to correctly identify the number of required doses remained associated with series completion (aRR 1.38; 95 % CI 1.08–1.76) [55]. Parents who remember receiving a provider recommendation for vaccination were more likely to have daughters who had completed the regimen (aOR 2.71; 1.99–3.70) [45]. However, general knowledge of HPV and HBV vaccine was not associated with completion in adjusted analyses [45, 47].

### Adverse events

Three studies assessed whether experience of adverse events following HPV vaccination affected series completion in the USA. Parents of daughters who had completed the three dose series reported pain or discomfort as often as parents whose daughters were late for their second or third dose (OR 0.76; 95 % CI 0.33–1.77) [61]. In a survey of over 3000 vaccine recipients [55] (response rate 27 %), multivariate analysis controlling for age, socio-economic status, health care utilization, showed reports of bruising or swelling at first dose did not affect completion of the series (aRR 0.88; 0.7–1.00). An association was not apparent for those reporting pain, syncope or dizziness [55]. A qualitative study of 18 women in the USA who did not complete the HPV vaccine series found none of them mentioned adverse events as a reason [62].

### Risk behaviour

A variety of risk behaviours in seven studies were assessed in relation to completion of HBV or HPV vaccine schedules; no associations were found. Drug use, history of sexually transmitted infections (STIs), or alcohol use was not associated with completion in the USA [38, 47, 63]. In multivariate analysis in Australia, intravenous drug use, sex work, or hepatitis C status did not correlate with likelihood of completion of HPV in a health unit serving at-risk populations [35].

### Concomittant healthcare

Three articles assessed the effect of concomitant health service delivery on adherence to HPV in the USA. Receipt of another vaccine at the time of HPV vaccination was not associated with odds of HPV completion controlling for socio-demographic and provider characteristics [55]. However, if the first dose was given at a health care provider visit which was attended primarily for another reason other than HPV, the odds of a mistimed 3rd dose were almost double (aOR 1.97; 95 % CI 1.39–2.80) than that if the first dose was at a vaccine only visit, controlling for age and race [48]. Type of visit was not associated in analysis investigating the effect of age and healthcare utilization [41].

### Access

Access to vaccination sites was assessed in two studies in the USA. Compliance to the schedule and completion of the series were not governed by proximity or mode of transportation to the clinical site [47]. Distance from home to clinic was not associated with completion controlling for age, race, and healthcare utilization [40].

### Qualitative studies

One qualitative study investigated why 9–26 year olds did not return for the final dose of the HPV vaccine series, in non-exclusive responses: 33 % claimed they didn’t know they were meant to obtain further doses, 23 % claimed they were too busy, 15 % cited inconvenience, 38.5 % claimed they were too busy or forgot, 7.7 % claimed they were too busy and times were inconvenient [62]. Two additional surveys of partially vaccinated university students in the USA and Australia indicated the potential problems with vaccinating older age groups who have competing priorities; reasons focused on inconvenience and lack of time [64, 65].

### Maternal characteristics

Three studies in the USA analyzed the relationship between maternal preventative behavior (cervical screening) and their daughter’s HPV vaccine series completion. In multivariate analysis, controlling for demographic, socioeconomic, family, and health plan characteristics, all three studies found that girls whose mothers had received a pap smear in the past three years were more likely to complete the HPV vaccine series (aOR 1.07, 95 % CI 1.06–1.08) [56]; 1.42, 1.31–1.54 [54] and 1.87, 1.31–2.75 [66]).

The relationship between maternal education and vaccine series completion was assessed in eight studies conducted in the USA (*n* = 7) and in Greece [51]. Adolescents whose mothers had less than high school education were less likely to complete the vaccine series in multivariate analysis [44, 66]; both studies controlled for adolescent age, SES, and mother’s health characteristics and found similar effect estimates (aOR 0.68; 95 % CI 0.56–0.84) [44]; aOR 0.60; 0.41–0.87 [66]). No association between maternal education and HPV or HAV vaccine series completion in multivariate analysis was found in three studies [39, 45, 50].

Maternal age and marital status were found to have no or very slight associations with vaccine series completion in four of the five included studies [39, 44, 45, 66]. In unadjusted analysis, one study found daughters with mothers aged over 40 years were more likely to complete the HPV vaccine series compared to mothers who were less than 40 years old [56].

### Provider/ organisational characteristics

#### Delivery model

There is strong evidence for high completion rates with school-delivery in high - income and low-middle income countries. Canadian in-school HPV vaccination completion rates were 75 % (95 % CI 74.7–75.8) compared to 36 % (95 % CI 35.3–37.2) for girls provided with a community-delivery model [58]. In-school vaccinations conferred 1.8 times the odds of completing the HBV series compared to if adolescents had to go off-site (95 % CI 1.15–2.8) in a parent survey in the USA controlling for age, race, insurance, SES, prior healthcare utilization [29].

Only 2 articles included data from low and middle - income countries (LMIC); descriptive results are available regarding the success of different delivery strategies [67]. In Uganda, a school-based strategy appeared more successful (94 % completion) than a strategy in which the vaccine was given in the community with a child health programme (79–87 % completion year 1-year 2) although the delivery strategies had slightly different target populations. Peru’s school-based strategy achieved 98.7 % completion, whilst combined school-based and health centre strategies in Vietnam achieved >99 % completion. In India, very similar completion rates were achieved in campaign and routine delivery approaches (97–98 %) [67]. Differences in completion rates achieved in 21 demonstration projects in 14 countries implementing different models of delivery were insignificant [68]; however, the mixed model (school based delivery with mop-up activities at health centres) seemed to confer marginally higher completion (96.6 %), the school-only model was intermediate (88.6 %) and the health facility-only model was least effective (79.7 %)(*p* = 0.39) [69].

In Australia, high-risk groups, benefited from an accelerated schedule (0, 7, 21 days and 12 months), which increased the likelihood of HBV vaccine completion 1.35 times (1.01–1.80) controlling for drug use, and length of contact with the health facility [35].

### Provider characteristics

Vaccine schedule completion was higher in an American school based programme when students returned the consent forms to their teacher compared to the school nurse [70]. In 1994–5 in Canada, initial parental consent was lower at private schools compared to public schools; however, private and public schools did not differ in completion rates. Different education providers (teachers or public health nurses) did not have an effect on completion, although education from teachers was associated with higher consent [30].

A further 17 studies investigated health provider characteristics, of which 12 reported adjusted analyses. There was no evidence that the speciality of an adolescent girl’s primary care physician influenced HPV series completion in multivariate analyses [15, 16, 36, 42, 49, 55]. However, for women >17 years of age in the USA, those with a paediatric/internal medicine physician were slightly less likely to complete the HPV regimen than those with a family medicine physician. Female providers were not significantly associated with completion (male primary care provider aRR0.93 0.85–1.01) [36]. In an American datalink study, those vaccinated at paediatric clinics had the highest completion (61 %) compared to family care practices (53 %; aOR 0.78; 95 % CI 0.76–0.80) and the local health departments (39 %; aOR 0.48; 0.47–0.50) [43].

## Discussion

We present a comprehensive review of the available literature on factors influencing adherence to multi-dose vaccine schedules among adolescents. The majority of studies took place in the USA (*n* = 49), the remainder included Canada (*n* = 3), France (*n* = 3), Australia (*n* = 2), Greece (*n* = 1), the UK (*n* = 1) and 2 multi-country studies including LMICs. The two studies including LMICs focused on organisational level factors and reported high adherence to HPV vaccine [68], therefore our summaries of individual level factors are limited in generalizability to developed settings. The high level of variation in the definitions, number and selection of factors investigated in each study limits the comparability of study results and prevented conduct of a meta-analysis. The overall impact of the identified characteristics on vaccine adherence is likely to be dependent on the mix of other factors present, as well as the programmatic and local context.

Good adherence to multi-dose vaccines appears to be higher in early adolescence (9–12 year olds) when compared to later adolescence (>14 years old). It is unclear whether this is linked to adolescent health seeking behaviour, which was inconsistently associated with completion, or whether it is governed by logistical reasons as cited in qualitative results. It could reflect factors which are not explored in the available literature such as which groups were most targeted with communication materials or the general decrease in utilization of health services through adolescence [71]. In some populations in the USA, there is evidence that Black or Hispanic girls are disproportionately prone to low completion rates when compared to White girls after adjustment for socioeconomic status and insurance, despite some reports of similar rates of initiation. Adolescent females may have a slightly elevated likelihood of vaccine completion compared to males; this association may be a symptom of increased opportunity whilst accessing contraception at the health centre. Higher household income, maternal education and maternal preventative health behaviour were associated with higher completion rates when compared to lower socio-economic families and those mothers who rarely sought screening. Insurance status may have a decreasing effect on completion over time as knowledge spreads that both HBV and HPV vaccines are eligible for reimbursement on any insurance plan in the USA. Experience of adverse events and general knowledge about the vaccine did not affect completion rates. School-based delivery alongside supplying vaccine to health centres for out-of-school girls appears to be a successful approach in countries with relatively high school attendance, including some LMICs [69], the UK [59] and Canada [58].

## Conclusions

The factors which affect rates of vaccine completion are context and time specific. Providers and programme planners should be aware that obtaining good consent and initiation rates is not enough; sub-groups within the population may need more help than others to complete the series. Efforts need to continue past the first dose to reduce inequality in completion. Adolescents captured for the first dose remain only partially protected from vaccine related disease until receipt of the final dose of the schedule.

Opportunistic vaccination at the delivery point of other services should be utilized as a strategy to increase vaccine completion. There is no evidence that concomitant service delivery is associated with lower completion. Among 11–18 year olds in Seattle, 71 % of visits to a primary health practitioner in 2006–11 were found to be lost opportunities for dose 3 of HPV vaccine [72]. Especially utilizing visits which were not originally for preventative health care services could rapidly improve completion rates and access those adolescents with low healthcare utilization [71, 72].

A Cochrane review in 2005 found 47 articles detailing the effect of patient reminder/recall on vaccine uptake, all were conducted in developed countries, only one study was conducted in adolescents [73]. In pooled results across all age groups, all reminder/recall systems appeared to improve coverage compared to the control groups. Personal telephone reminders were the most effective intervention (OR 1.92; 95 % CI 1.2–3.07). Letter reminders were close to the effectiveness of phone reminders (OR 1.79; 1.5–2.15), a postcard alone was less effective (OR 1.44; 1.09–1.89), and automated phone calls were least effective (OR 1.29; 1.09–1.53). Interventions to improve completion of vaccine series need to be assessed and the use of novel technologies needs to be explored where electronic records and recall systems are not available.

## Additional file

Additional file 1: Table S1. Search terms and results Medline (Ovid SP)1946 to 2014-02-26. (DOCX 17 kb)
