# Supplementary material for: Factors influencing completion of multi-dose vaccine schedules in adolescents: a systematic review
Source: BMC Public Health. 2016 Feb 19;16:172. doi: 10.1186/s12889-016-2845-z (PMC4759915; doi:10.1186/s12889-016-2845-z)
Supplement: Additional file 1: Table S1. — Search terms and results Medline (Ovid SP)1946 to 2014-02-26. (DOCX 17 kb) [file 12889_2016_2845_MOESM1_ESM.docx]

**Additional file 1: Table S1.** Search terms and results Medline (Ovid SP)1946 to 2014-02-26.

*Caption: An example of the search terms used and the number of hits from Medline database (Ovid SP)*

| **Adolescent vaccine adherence** | | **Medline(Ovid SP) 1946 to 2013-10-28** | **Results** |
| --- | --- | --- | --- |
| **Search** | **Item** | **Synonyms** |  |
| 1 | textword | school or young adult$ or youth or boy$ or girl$ or schoolage$ or (school adj1 age$) or schoolchild$ or primary school$ or elementary school$ or prepubescen$ or secondary school$ or pubescen$ or adolescen$ or juvenil$ or teen or teens or teenage$ or (youth or youths) or (highschool$ or (high adj1 school$)) or offspring | 2,099,537 |
| 2 | Subject heading | Exp Adolescent/  Exp adolescent health services/  Exp adolescent medicine/  Exp young adult/ |  |
| 3 | Journal titles | adolescen$.jw. or youth$.jw. or school$.jw. | 43,090 |
| 4 | 1 or 2 or 3 |  | 2,112,749 |
| 4 | Textword | Vaccine* or vaccination*or immunis* or immuniz* or human papillomavirus vaccin* or HPV vaccin* or Cervarix or Gardasil or meningococcal vaccin* or Meningitis-A vaccin* or meningococcal-A or MenAfriVac or Meningococcal A+C or POLYSACCHARIDE MENINGOCOCCAL A+C VACCIN* or Hib-MenCY or MCV4-D or MCV4-CRM or Menomune or Meningococcal ACYW-135 Polysaccharide vaccin* or Hepatitis-A vaccin* or HepA vaccin* or Havrix or Hepatitis-B vaccin* or HepB vaccin* or Hep-B vaccin* or Hepatitis-B recombinant or hepatitis-B vaccine recombinant or Euvax B or Engerix-B or Heberbiovac HB or Hepavax-Gene TF or Hepavax-Gene or Shanvac-B | 327,567 |
| 5 | Subject heading | Exp Vaccination/  Exp Immunization programs/  Exp Papillomavirus vaccines/  Meningococcal Vaccines/  Exp viral hepatitis vaccines/ | 66,191 |
| 6 | 4 or 5 |  | 324,475 |
| 7 | Textword | Coverage or uptake or continuation or completion or adherence or compliance or dropout*or drop-out* or retention | 586,046 |
| 8 | Subject heading | Exp patient compliance/ | 55,248 |
| 9 | 7 or 8 |  | 701,239 |
| 10 | 3 and 6 and 9 |  | **4,762** |
